# Small Cell Neuroendocrine Carcinoma of the Ureter: A Case Evaluated by ^18^F-FDG-PET/CT and Literature Review

**DOI:** 10.2174/0115734056353458250429063852

**Published:** 2025-05-13

**Authors:** Rong Yang, Liqin Gu, Chengzhou Li, Qiong Song, Yanfang Bao, Lan Lin, Juan Chen

**Affiliations:** 1 The Department of Nuclear Medicine, Tongren Hospital, Shanghai Jiao Tong University School of Medicine, Shanghai, China; 2 The Department of Pathology, Tongren Hospital, Shanghai Jiao Tong University School of Medicine, Shanghai, China

**Keywords:** PET/CT, small cell neuroendocrine carcinoma, ureter, Intensive tracer activity, Chemotherapy

## Abstract

**Introduction:**

Small cell neuroendocrine carcinoma (SCNEC) of the ureter is extremely rare, and tends to show a mixed histologic profile. Literature on its imaging features is limited.

**Case Presentation:**

We herein report the case of a 68-year-old woman who presented with two days of left flank pain. Ultrasound and CT scan revealed a lesion in the left distal ureter. The lesion exhibited intensive tracer activity on ^18^F-FDG PET/CT scan, corresponding to a malignant tumor, most likely a high-grade urothelial carcinoma, and no metastases were observed. Then, the patient underwent a radical left nephroureterectomy. Pathology revealed a carcinoma composed of SCNEC (approximately 83%) and urothelial carcinoma (approximately 17%). During one year of follow-up, the patient underwent six cycles of adjuvant chemotherapy (etoposide 100mg d1-3 + cisplatin 30mg d1-3, q3w), and no recurrence or metastases were found on the CT scan.

**Conclusion:**

This case report has presented a case of ureteral SCNEC and explored the value of ^18^F-FDG PET/CT in the diagnosis and staging of the disease.

## 1 INTRODUCTION

Neuroendocrine tumors (NETs) commonly arise from the lung, the gastrointestinal tract, and the pancreas [1], with or without neuroendocrine function. Ureteral NETs are rare with only around 30 cases reported in the literature [2], and ureteral small cell neuroendocrine carcinomas (SCNECs) are especially rare with limited case reports.

Here, we present a case of ureteral SCNEC. To the best of our knowledge, this is the first case of this location evaluated by ^18^F-FDG PET/CT after reviewing the English literature.

## 2 CASE REPORT

A 68-year-old woman presented to the hospital with a complaint of left flank pain for two days. She was healthy except for a previous medical history of subtotal thyroidectomy for papillary thyroid cancer two years ago. The patient was not a smoker or drinker, and denied any abnormal childbearing history.

The urinalysis disclosed many positive findings, such as increased urinary protein, erythrocytes, and leukocyte esterase. Serum creatinine level was elevated to 108.5 umol/L (with a normal range of 46 umol/L to 92 umol/L), corresponding to an estimated glomerular filtration rate of 34.5 mL/min/1.73m^2^. Tumor marker tests of serum, including carbohydrate antigen 50 (CA50), CA242, CA153, CA199, CA125, CA724, alpha-fetoprotein (AFP), carcinoembryonic antigen (CEA), chorionic gonadotropin (hCG), cytokeratin 19 fragment (cyfra211), neuron-specific enolase (NSE), progastrin-releasing peptide (ProGRP), and squamous cell carcinoma-associated antigen (SCCAg), were all normal, and a few atypical epithelial cells were detected on urine cytology.

**Fig. (1):**
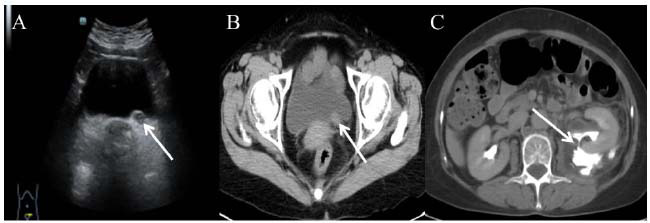
Ultrasound and CT scan of the lesion. Trans-abdominal ultrasound showed a hypoechoic lesion in the left distal ureter (**A**, arrow). The CT scan showed a lesion (**B**, arrow), accompanied by left hydronephrosis and rupture of the renal pelvis (**C**, arrow).

**Fig. (2):**
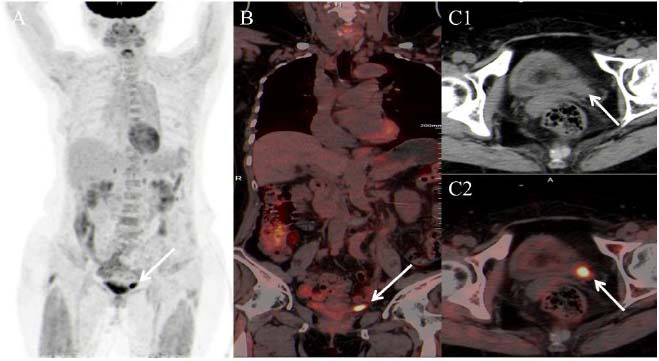
The lesion shows clearly with intensive metabolic activity (SUVmax= 12.64) on ^18^F-FDG PET/CT scan (arrows). (**A**) Maximum intensity projection image; (**B**) Coronal fusion image; (**C1**) Transverse CT plain scan image; (**C2**) Transverse fusion image).

Regarding the imageological examinations, an ultrasound examination was conducted first. It revealed severe left hydronephrosis, and an associated hypoechoic material with no Doppler flow was discovered in the distal part of the left ureter, potentially debris or a mass (Fig. **1A**). Subsequently, a CT urography scan was conducted for detailed characterization of the lesion. It demonstrated a 20mm soft tissue lesion (Fig. **1B**), accompanied by left hydronephrosis and rupture of the renal pelvis (Fig. **1C**). In consideration of her previous history of papillary thyroid cancer, an ^18^F-FDG PET/CT scan was performed to assess the lesion after left percutaneous nephrostomy. Due to the absence of urine in the left ureter, the lesion was visualized clearly with intensive metabolic activity (standard uptake value, SUVmax = 12.64). Except for some non-specific radiopharmaceutical uptakes, no other abnormal radiopharmaceutical uptakes were identified, so the lesion was considered to be a primary malignant tumor, most likely a high-grade urothelial carcinoma (Fig. **2**). Based on these observations, a radical left nephroureterectomy was recommended. Because urothelial carcinoma of the ureter tends to implant in the bladder, a cystoscopy was performed preoperatively, and it showed a normal bladder. The operation was uneventful, and the patient was discharged on postoperative day 8. One month later, she was transferred to another hospital for adjuvant chemotherapy (etoposide 100mg d1-3 + cisplatin 30mg d1-3, q3w, six cycles) without any adverse or unexpected events. She was followed up for one year without recurrence or metastases on the CT scan.

**Fig. (3):**
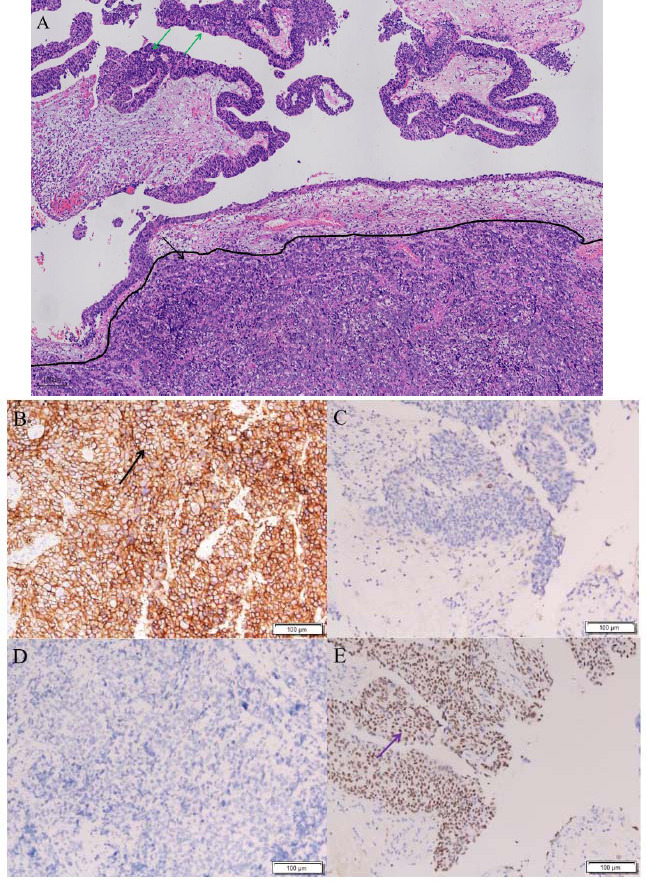
Hematoxylin eosin stained image of the lesion. The majority (approximately 83%) were small-sized round cells with hyperchromatic nuclei (black arrow), while the remainder (approximately 17%) were urothelial carcinoma *in situ* cells (green arrows) (**A**). The small-sized round cells were strongly positive for the marker CD56 (**B**, arrow) and negative for the marker GATA3 (**D**), and the urothelial carcinoma *in situ* cells were negative for the marker CD56 (**C**) and positive for the marker GATA3 (**E**, arrow).

A gross histopathological examination disclosed a gray-white and moderate texture mass in the left distal ureter. Hematoxylin and eosin stain showed that the mass consisted of two types of tumor cells. The majority were small-sized round cells (about 83%) with hyperchromatic nuclei, exhibiting positivity for Brg1, CD200, CD56, INSM1, and synaptophysin, but negativity for CK7, CK20, CK5/6, chromogranin, CD30, GATA3, and CD20. The Ki67 proliferation index was 80%. The rest cells were urothelial carcinoma *in situ* (about 17%), positive for CK7, CK20, GATA3, CK5/6, Brg1, CD30, and CD20, but negative for CD200, chromogranin, synaptophysin, CD56, and INSM, with a Ki67 index of 40%. DAXX and ATRX mutations were not tested. According to the immunohistochemical staining results, especially the strong positive expression for the marker CD56 [3], the small-sized round cells were diagnosed as poorly differentiated SCNECs (Fig. **3**). The tumor infiltrated the superficial muscle layer of the ureter with vascular invasion and nerve invasion, and the tumor stage was T2N0M0.

## 3 DISCUSSION

Neuroendocrine tumors (NETs) of the urinary tract account for less than 0.05% of primary urinary tract malignancies, and most originate in the bladder [4-7] and prostate [8, 9]. A short review including 28 patients indicated ureteral NETs to have a male predominance (about 70.4%), and most were found to present after the sixth decade of life (mean age: 68.25 years; range: 48 to 86 years) [4]. However, they can also occur at a younger age [10].

Clinical presentations of ureteral NETs are similar to those of more common ureteral neoplasms [10]. If based on ultrasound and CT findings only, benign ureteral neoplasms, such as papilloma, should also be considered. However, given the intensive FDG uptake of the lesion, a malignant tumor should be considered first, most likely a high-grade urothelial carcinoma. Granulomatous diseases, such as IgG4-related disease, may also show intensive FDG uptake during the active phase, but IgG4-related disease tends to involve multi-organs and rarely involve the ureter alone [11]. For the above reasons, the case was initially diagnosed as a high-grade urothelial carcinoma, but the pathological examinations indicated it as a case of SCNEC with concomitant focal urothelial carcinoma *in situ*. When we analyzed the case retrospectively, we noticed that small-sized urothelial carcinomas rarely show such intensive FDG uptake, and that ^18^F-FDG PET/CT played an important role in the qualitative diagnosis and tumor staging in this case.

The diagnosis of SCNEC depends mainly on pathology. SCNECs are typically small to medium-sized, round to spindle-shaped cells with negligible cytoplasm and hyperchromatic nuclei [2]. Immunohistochemically, they can be positive for chromogranin, CD56, synaptophysin, and neuron-specific enolase [2]. They usually present a very high nuclear Ki-67 index and show intense radiopharmaceutical uptake on ^18^F-FDG PET/CT. Over half of ureteral SCNECs show a mixed histologic profile [4], as was the case in our study.

NETs are clinically heterogeneous, and their treatment depends on many factors, including pathological grading, clinical staging, tumor location, and so on [12]. Compared to other NETs, SCNECs are often diagnosed at a later stage due to their aggressive nature. Therefore, surgery is often not advisable, and the prognosis of SCNEC is poor, especially in advanced cases [13, 14]. Given the rarity of ureteral SCNECs, there is no recommended standard treatment. Surgery, chemotherapy, radiotherapy, and their combinations have all been reported [3, 14, 15]. The available literature supports neoadjuvant cisplatin-based chemotherapy, followed by nephroureterectomy [3].

## CONCLUSION

This case showed that SCNEC should be considered in the differential diagnosis of a ureteral mass when it shows intensive radiopharmaceutical uptake. ^18^F-FDG PET/CT is useful for diagnosing and staging SCNEC, as it shows intense radiopharmaceutical uptake for a high proliferative index.

## AUTHORS’ CONTRIBUTIONS

The authors confirm their contribution to the paper as follows: study conception and design: JC, RY; data collection: LQG, LL; analysis and interpretation of results: QS, CZL, YFB, LL; drafting of the manuscript: JC, RY. All authors have reviewed the results and approved the final version of the manuscript.

## Data Availability

The data and supportive information are available within the article.

## LIST OF ABBREVIATIONS

NET: Neuroendocrine tumor
SCNEC: Small cell neuroendocrine carcinoma
SUV: Standard uptake value

## STANDARDS OF REPORTING

CARE guidelines were followed.
